# The Prevalence of Cardiovascular Risk Factors in Patients With Diabetes Mellitus in Yemen: A Systematic Review and Meta-Analysis

**DOI:** 10.7759/cureus.72848

**Published:** 2024-11-01

**Authors:** Sami Al-Hajj, Zahid Khan, Amresh Gul, Osman Ahmed, Animesh Gupta, Gideon Mlawa

**Affiliations:** 1 Cardiology, University of South Wales, Pontypridd, GBR; 2 Acute Medicine, Mid and South Essex NHS Foundation Trust, Southend on Sea, GBR; 3 Cardiology, Bart’s Heart Centre UK, London, GBR; 4 Cardiology and General Medicine, Barking, Havering and Redbridge University Hospitals NHS Trust, London, GBR; 5 Cardiology, Royal Free Hospital, London, GBR; 6 General Practice, General Practice Clinic, Brisbane, AUS; 7 Internal Medicine, Mid and South Essex NHS Foundation Trust, Southend on Sea, GBR; 8 Acute Internal Medicine, Southend University Hospital NHS Trust, Southend on Sea, GBR; 9 Acute Internal Medicine/Intensive care, Barking, Havering and Redbridge Hospital NHS Trust, London, GBR; 10 Internal Medicine and Diabetes and Endocrinology, Barking, Havering and Redbridge University Hospitals NHS Trust, London, GBR

**Keywords:** american heart association (aha) and american college of cardiology (acc), a systematic review and meta-analysis, community obesity, coronary heart disease (chd), family history of diabetes, hyperlipidemia treatment, hypertension and therapy, newcastle-ottawa-scale, prevalence of smoking, risk factors of cardiovascular diseases

## Abstract

Cardiovascular disease (CVD) is a major global health challenge, and its co-occurrence with diabetes mellitus (DM) contributes significantly to morbidity and mortality. Yemen, a nation facing unique healthcare complexities, necessitates an in-depth investigation into the prevalence of cardiovascular risk (CVR) factors among its population with DM. This systematic review and meta-analysis aimed to determine the prevalence of CVR factors among individuals with diabetes in Yemen, to understand and highlight the knowledge gap, and to influence targeted interventions and policies. This systematic review followed the Preferred Reporting Items for Systematic Reviews and Meta-Analyses (PRISMA) guidelines. A comprehensive literature search was conducted across PubMed, Embase, Scopus, Google Scholar, and African Journal Online (AJOL) databases. Medical subject headings (MeSH) terms and keywords related to DM, CVR factors, Yemen and prevalence were used. Studies published from inception to December 2020 were considered, with inclusion criteria including all Yemeni diabetic patients older than 18 years, and studies conducted in Yemen that provided sufficient data to calculate their corresponding confidence intervals. Data extraction and quality assessment were conducted independently by two investigators. This study provides a comprehensive overview of the prevalence of CVR factors among individuals with DM in Yemen. A meta-analysis was conducted to estimate pooled prevalence rates using random-effects models. This systematic review and meta-analysis aimed to address the scarcity of data on CVR factors among individuals with DM in Yemen. By synthesizing existing evidence, this research seeks to enhance our understanding of the cardiovascular health landscape in Yemen and provide crucial insights for healthcare interventions. These findings will contribute valuable evidence to inform strategies aimed at reducing CVD-related morbidity and mortality among Yemeni individuals with DM.

## Introduction and background

Yemen is located near the southwestern tip of the Arabian Peninsula in Southwest Asia. Sprawling over approximately 523 km2, it is regarded as the second most expansive nation on the Peninsula [1]. Yemen’s total population is estimated to be 3.4 million people in 2022, according to the most recent United Nations estimates. Yemen’s pyramidal age structure indicates that the young represent most of the population [2]. It is one of the poorest nations, ranking 177th in the world in terms of Human Development Index (HDI) [3]. According to the global burden of disease, contagious diseases, newborns, and nutritional disorders (account for half of all fatalities in Yemen) are the leading causes of preventable deaths, followed by non-contagious diseases [4].

Diabetes mellitus (DM) refers to a disorder characterized by hyperglycemia caused by improper glucose metabolism. It is related to a relative or absolute decrease in insulin production, as well as various degrees of peripheral insulin resistance. Type 2 diabetes is the most prevalent form of diabetes among adults (>90%) [5,6]. The traditional indications of hyperglycemia, such as excessive urination and increased thirst, impaired vision, and reduction in body weight, are frequently observed only in hindsight after the identification of an elevated blood glucose level [7,8].

The Incidence of DM has increased substantially over the last several decades. Approximately 8.8 percent of the adult global population, or more than 400 million individuals, were diagnosed with DM in 2017. That number is projected to increase to almost 650 million by the year 2040 [9].

Hypertension is a leading modifiable risk factor and a major factor for cardiovascular diseases (CVDs) among both diabetic and non-diabetic patients. Diabetic patients tend to have twice the prevalence of hypertension compared to non-diabetic patients, as reported by previous epidemiologic studies. Hypertension is the most common condition of CVD found in diabetic patients [10,11,12].

Tobacco smoking significantly contributes to the increased risk of CVD and DM. Tobacco smoke contains hazardous compounds, including carbon monoxide, nicotine, and oxidant gases, which may potentially contribute to the development of CVDs [13,14]. CVDs are induced by tobacco via several pathophysiological mechanisms. These include complications such as inflammation, platelet aggregation, insulin resistance, dyslipidemias, hypercoagulability, and damage to artery lining integrity [15,16].

Obesity significantly elevates the likelihood of developing type 2 DM, hypertensive disease, stroke, and several other complications. Adults are considered overweight if their BMI is equal to or more than 25, and obesity is diagnosed with a BMI of 30 kg/m2 [17,18]. Impaired glucose tolerance and type 2 diabetes are conditions that become more probable as body mass increases. Both diabetes and obesity have similar pathogenesis and share common pathways of oxidative stress, lipid metabolism, insulin resistance, pro-inflammatory, and pro-thrombotic patterns [19,20].

Hyperlipidemia, an essential risk factor for atherosclerosis and coronary heart disease (CHD), is often induced as a secondary consequence of DM, especially when glycemic control is inadequate. Insulin resistance or insufficiency impacts critical enzymes and pathways in lipid metabolism, which contributes to the prevalence of lipid abnormalities in DM [21].

Patients who have a first-degree relative with a familial history of type 2 diabetes have a two to threefold greater chance of having the condition, compared to those without such a familial history. Those with a familial and maternal history of type 2 diabetes have a fivefold to sixfold increased chance of developing the disease. Potential mediators of the risk include lifestyle variables such as food, physical activity, and smoking, as well as anthropometric measurements like BMI and waist circumference [22-25].

## Review

Aims and objectives

This review aimed to systematically determine the prevalence of CV risk factors (hypertension, smoking, obesity, hyperlipidemia, and family history) among individuals with DM in Yemen, in addition to determining the regional distribution of these risk factors facilitating a better understanding of the evolving cardiovascular landscape within the Yemeni population.

Ethical consideration

We received ethical approval from the University of South Wales research ethics committee. Given the utilization of secondary data, the need for participant informed consent will be appropriately waived.

Methodology

This was a systematic review and meta-analysis of original studies that reported the prevalence of CV risk factors (hypertension, smoking, obesity, hyperlipidemia, and family history) among individuals with DM in Yemen.

We systematically searched databases including PubMed, Scopus, Google Scholar, Web of Science, African Journal Online (AJOL), and the Cochrane Library for studies on the epidemiology of any of CV risk factors (hypertension, smoking, obesity, hyperlipidemia, and family history) among individuals with DM in Yemen.

We imported all the studies obtained from the literature search to our database using EndNote® (Clarivate, Berkeley, CA) software, and duplicates were removed using the same software. All articles were screened for inclusion by using their titles and abstracts to assess their relevance to our study question. The full-text articles of the relevant studies were obtained for further review after the exclusion of the irrelevant articles that did not meet our inclusion criteria.

Inclusion Criteria

Studies were included in this review if they met the following inclusion criteria. Studies involving Yemeni diabetic patients older than 18 years, studies conducted in Yemen providing sufficient data to calculate their corresponding confidence intervals, and studies published in the English language among diabetic patients. There were no limitations applied in terms of duration.

Exclusion Criteria

We excluded studies involving patients younger than 18 years, studies not conducted in Yemen, studies without baseline data, and studies published in non-English language.

Data Extraction

After obtaining full-text articles that meet the inclusion criteria, we extracted data relating to the study’s authors, publication year, study design, region, sample characteristics, total sample size, age distribution, gender distribution, and duration of DM diagnosis. Additionally, we also extracted data related to cardiovascular risk (CVR) factors such as the prevalence of hypertension, diabetes, dyslipidemia, obesity, and cigarette smoking among individuals with DM.

The extracted data were captured in Microsoft Excel® by using data abstraction form. The Preferred Reporting Items for Systematic Reviews and Meta-Analyses (PRISMA) protocol was used for this systematic review and meta-analysis.

**Figure 1:**
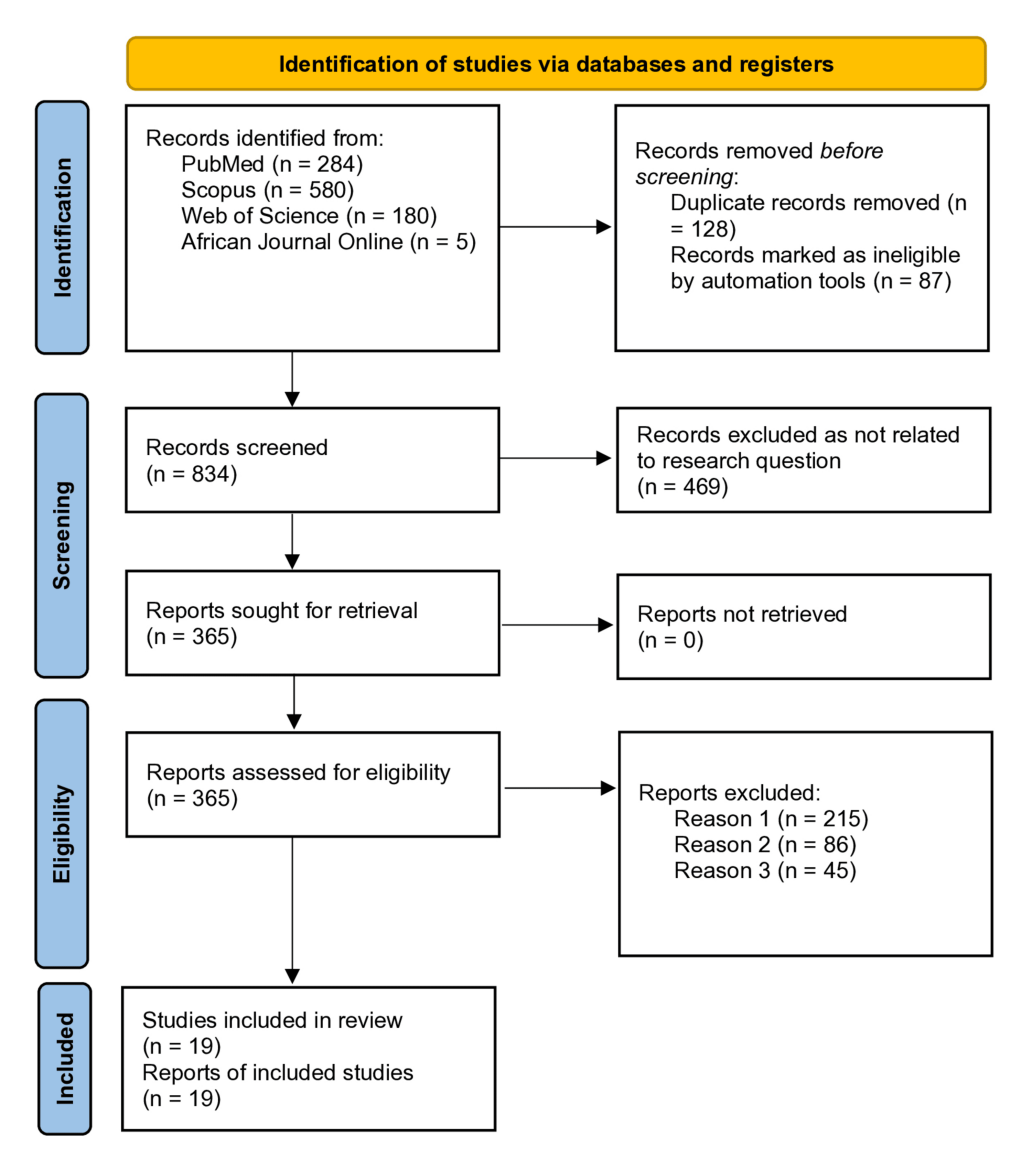
PRISMA 2020 flow diagram for the systematic review and meta-analyses. PRISMA: Preferred Reporting Items for Systematic Reviews and Meta-Analyses The flow diagram was prepared by author Dr. Zahid Khan using RevMan software.

We used medical subject headings (MeSH) terms for the literature search and obtained a total of 1049 articles (PubMed: 284, Scopus: 580, Web of Science (WoS): 108 and African Journal Online: 5). The detailed breakdown of the included studies is presented in the PRISMA flow diagram (Figure 1).

Risk of Bias Assessments

**Figure 2:**
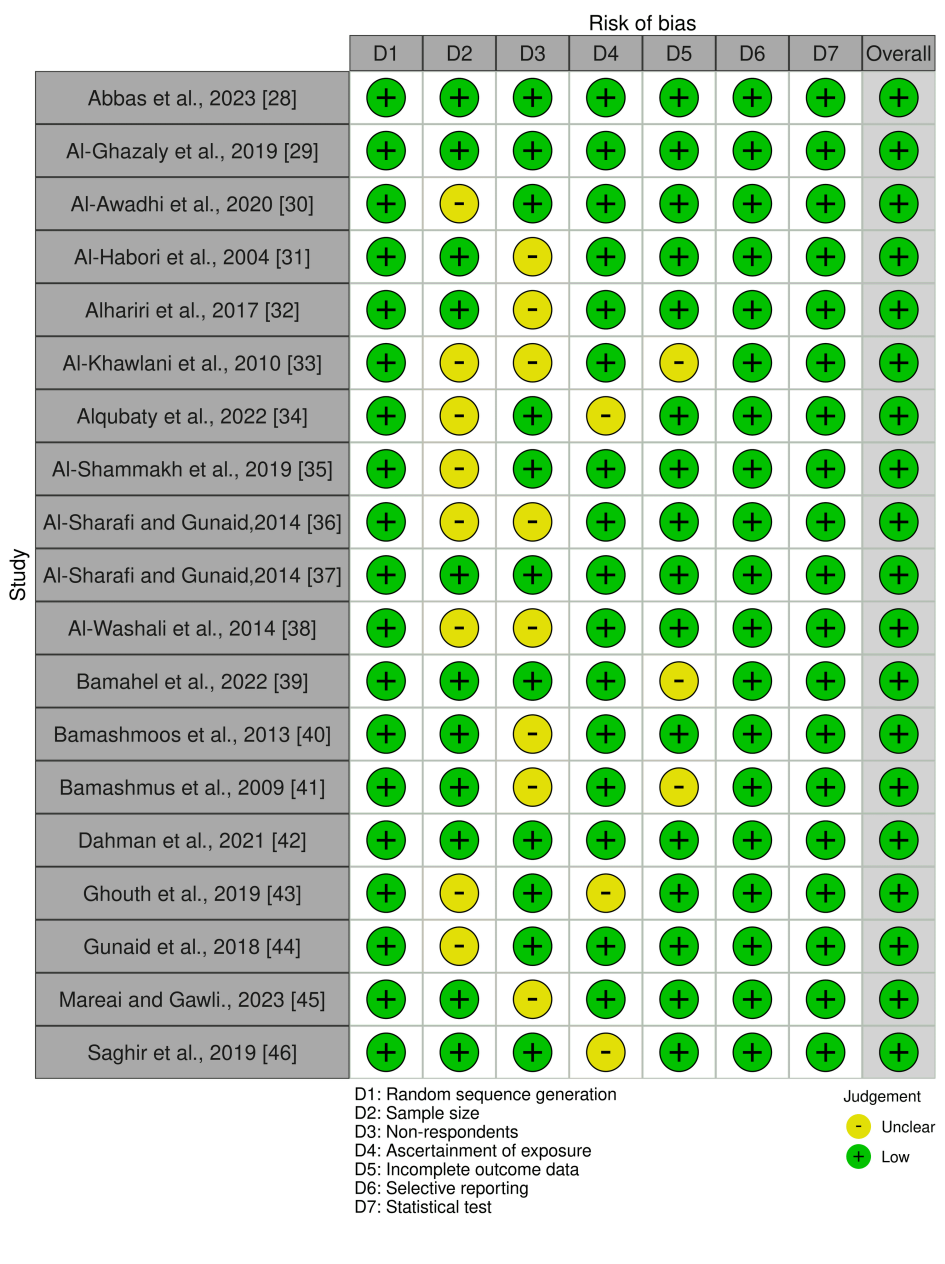
Traffic-light plot for the risk of bias assessment for included studies. The image was prepared by author Dr. Zahid Khan using RevMan software.

**Figure 3:**
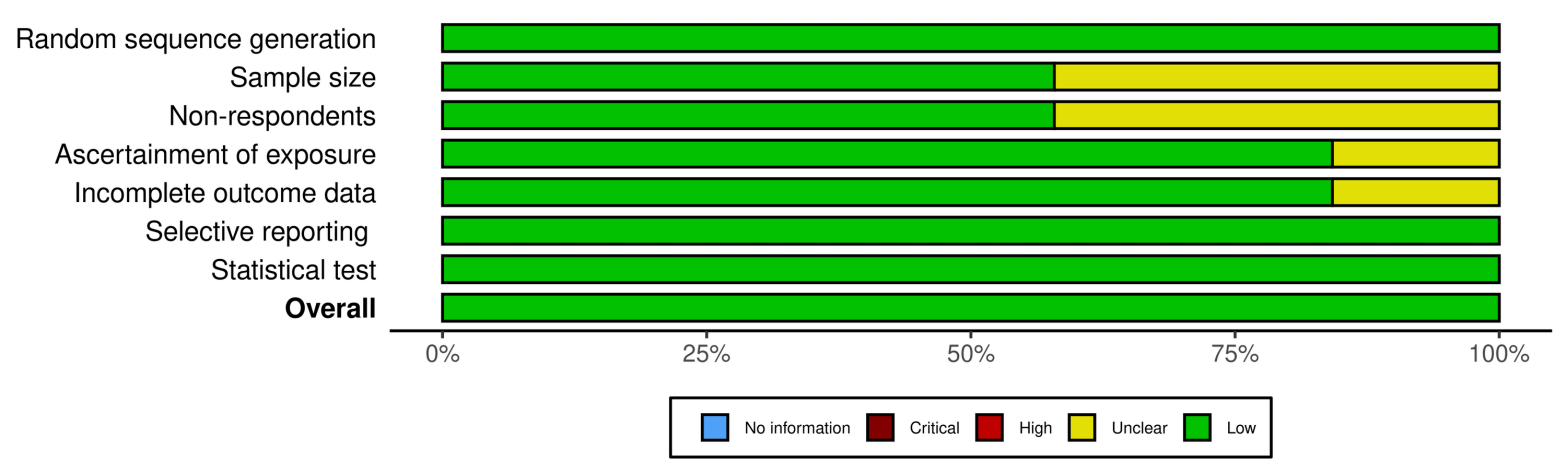
Summary plot for the risk of bias assessment for included studies. The image was prepared by author Dr. Zahid Khan using RevMan software.

The Newcastle-Ottawa-Scale (NOS) and a modified version of NOS by Herzog et al., were used to evaluate the quality of case-control and prospective cohort studies and cross-sectional studies, respectively [26,27]. Both scoring systems examined the quality of studies based on the comparability of the studied groups, the selection of the cohort population, and the ascertainment of either the exposure or outcome of interest for case-control or cohort studies. A maximum of 9 points for prospective cohort and case-control studies and 10 points were given to cross-sectional studies and studies scoring 6 or above were considered to be at low risk of bias. All the included studies had a low risk of bias based on the NOS and modified NOS scales that were used to evaluate the quality of case-control and prospective cohort studies and cross-sectional studies (Figures 2-3).

Statistical Methods

The collected data was analyzed thoroughly using Cochrane Review Manager Software version (RevMan) 5.4.0. Data heterogeneity was evaluated by employing the Q statistic (I2) in conjunction with its corresponding p-value. The extent of heterogeneity was categorized as low (I2 = 0%-25%), moderate (I2 = 26%-50%), or high (I2>50%). The choice of statistical models was contingent upon the observed degree of heterogeneity: random-effect models will be applied when I2≥50%, while fixed-effect models will be employed for cases of I2<50%. These models were used to determine the prevalence of CV risk factors among individuals with DM in Yemen presented alongside their corresponding 95% confidence intervals. The outcomes of the meta-analysis were visually represented using a forest plot. We assessed the presence of publication bias using funnel plots.

Results

The sample size of the participants in the studies ranged from 23 to 1640 participants, with a total of 7141 participants. The mean age of participants ranged from 47 to 56 years with a weighted mean age of 50.7 years. The female proportion ranged from 22 to 65% with a weighted mean proportion of 52.1%. The mean duration of DM ranged from 4.5 to 10.6 years with a weighted mean duration of 6.6 years. Seven of the included studies were carried out in Sana’a, the capital of Yemen, three in Mukalla city, two in Ibb city, two in Dhamar city, and two in Hodeidah city. Three studies didn’t report the region where they were carried in. Of the included articles, there were 17 cross-sectional studies and 2 case-control studies.

The included studies were carried out in tertiary hospitals and clinics. Participants in all these original studies had face-to-face interviews and data collection were done in these studies by the authors using an Arabic structured questionnaire and laboratory data were measured during the time of interviewing. The characteristics of the 19 studies included in the meta-analysis are summarized in Table 1.

**Table 1:** Characteristics of the 19 studies’ population included in the systematic review and meta-analysis showing demographic characteristics

Author	Study design	Region	Sample size	Female proportion	Age (years)	Duration of DM (years)	Proportion diagnosed with hypertension	Proportion of smokers	Proportion of obese	Proportion diagnosed with hyperlipidemia	Proportion with family history
Abbas et al., 2023 [28]	Case-control study	Ibb	102	51%	48.61 ± 14.8	6.5 ± 6.3	-	16.67%	-	-	62.75%
Al-Ghazaly et al., 2019 [29]	Cross-sectional study	Sana’a	324	57%	53 ± 12	4.5	48.80%	-	31.48%	56.48%	-
Al-Awadhi et al., 2020 [30]	Cross-sectional study	Ibb	100	65%	50.9 ± 12.8	-	-	22.50%	-	-	-
Al-Habori et al., 2004 [31]	Cross-sectional study	Sana’a	23	22%	48.2 ± 11.3	-	21.74%	-	9.09%	34.78%	-
Alhariri et al., 2017 [32]	Cross-sectional study	Hodeidah	210	45%	47.6 ± 12.6	≤5:51%	-	28.10%	-	-	-
						>5: 49%					
Al-Khawlani et al., 2010 [33]	Cross-sectional study	Sana’a	311	50%	53.9 ± 10.9	6.3 ± 6.5	53.40%	21.20%	8.70%	-	50.80%
Alqubaty et al., 2022 [34]	Cross-sectional study	Sana’a	100	42%	47.9 ± 11.7	6.7 ± 5.2	-	-	19.00%	-	67.00%
Al-Shammakh et al., 2019 [35]	Cross-sectional study	Dhamar	200	43%	≥ 50: 52%	<5:15%	32.00%	27.50%	47.00%	68.50%	43.00%
					< 50: 48%	5-10:56%					
						>10: 29%					
Al-Sharafi and Gunaid, 2014 [36]	Cross-sectional study	-	1640	56%	50.3 ± 11.5	5.0 ± 6.0	-	-	16.83%	-	-
Al-Sharafi and Gunaid, 2015 [37]	Cross-sectional study	-	1540	56%	49.6	≥10:22.3%	29.10%	20.20%	24.74%	-	-
						<10: 77.7%					
Al-Washali et al., 2014 [38]	Cross-sectional study	Sana’a	306	54%	53.5 ± 11.1	7.14 ± 5.7	27.45%	-	25.49%	-	-
Bamahel et al., 2022 [39]	Cross-sectional study	Mukalla	127	47%	52.18	<5:41.7%	40.90%	-	-	-	-
						5-10:30.7%					
						11-15:13.4%					
						>15: 14.2%					
Bamashmoos et al., 2013 [40]	Cross-sectional study	Sana’a	500	56%	56.3 ± 32	10.6±54	51.00%	26.00%	19.60%	-	-
Bamashmus et al., 2009 [41]	Cross-sectional study	Sana’a	350	40%	-	<5: 32.3%	23.14%	-	-	-	-
						5 to 9: 15.1%					
						10 to 14: 23.7%					
						>15: 28.9%					
Dahman et al., 2021 [42]	Case-control study	Mukalla	142	55%	54.0 ± 8.29	-	31.70%	7.75%	24.60%	-	31.70%
Ghouth et al., 2019 [43]	Cross-sectional study	Mukalla	120	51%	54.81 ± 9.33	-	-	-	25.00%	85.00%	-
Gunaid et al., 2018 [44]	Cross-sectional study	-	500	37%	47 ± 11	-	35.00%	28.20%	31.20%	28.00%	54.40%
Mareai and Gawli, 2023 [45]	Cross-sectional study	Dhamar	300	56%	30–50: 39%	≤5:55.3%	35.66%	-	23.00%	-	-
					>50: 61%	>5: 44.7%					
Saghir et al., 2019 [46]	Cross-sectional study	Hodeidah	246	48%	49.5 ± 11.8	<7:65%	45.90%	29.30%	16.70%	61.00%	-
						≥7: 35%					

Prevalence of hypertension

**Figure 4:**
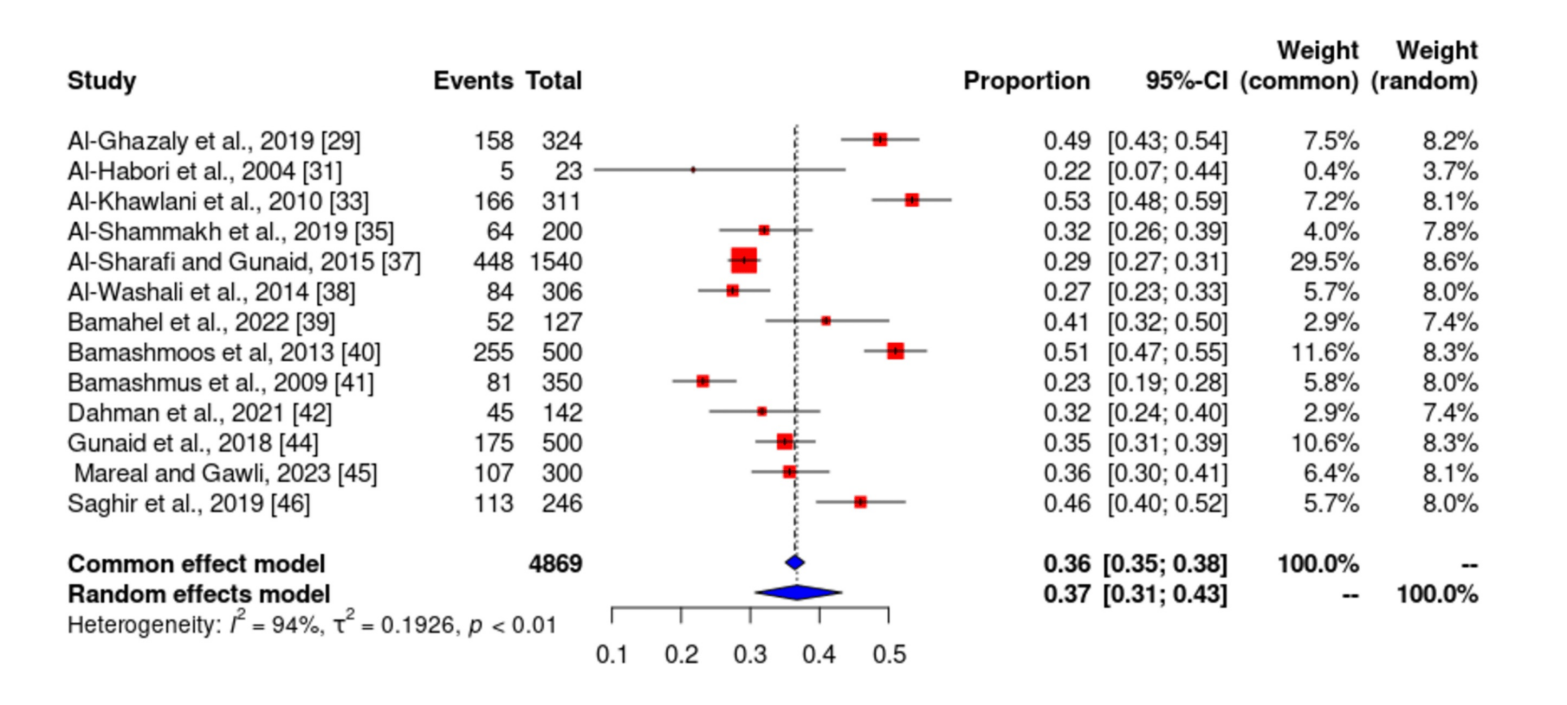
The prevalence of hypertension among diabetes mellitus patients in Yemen. The red boxes represent the effect estimates (prevalence) provided as a percentage. The diamond is the pooled effect estimate at a 95% confidence interval. The image was prepared by author Dr. Zahid Khan using RevMan software.

**Figure 5:**
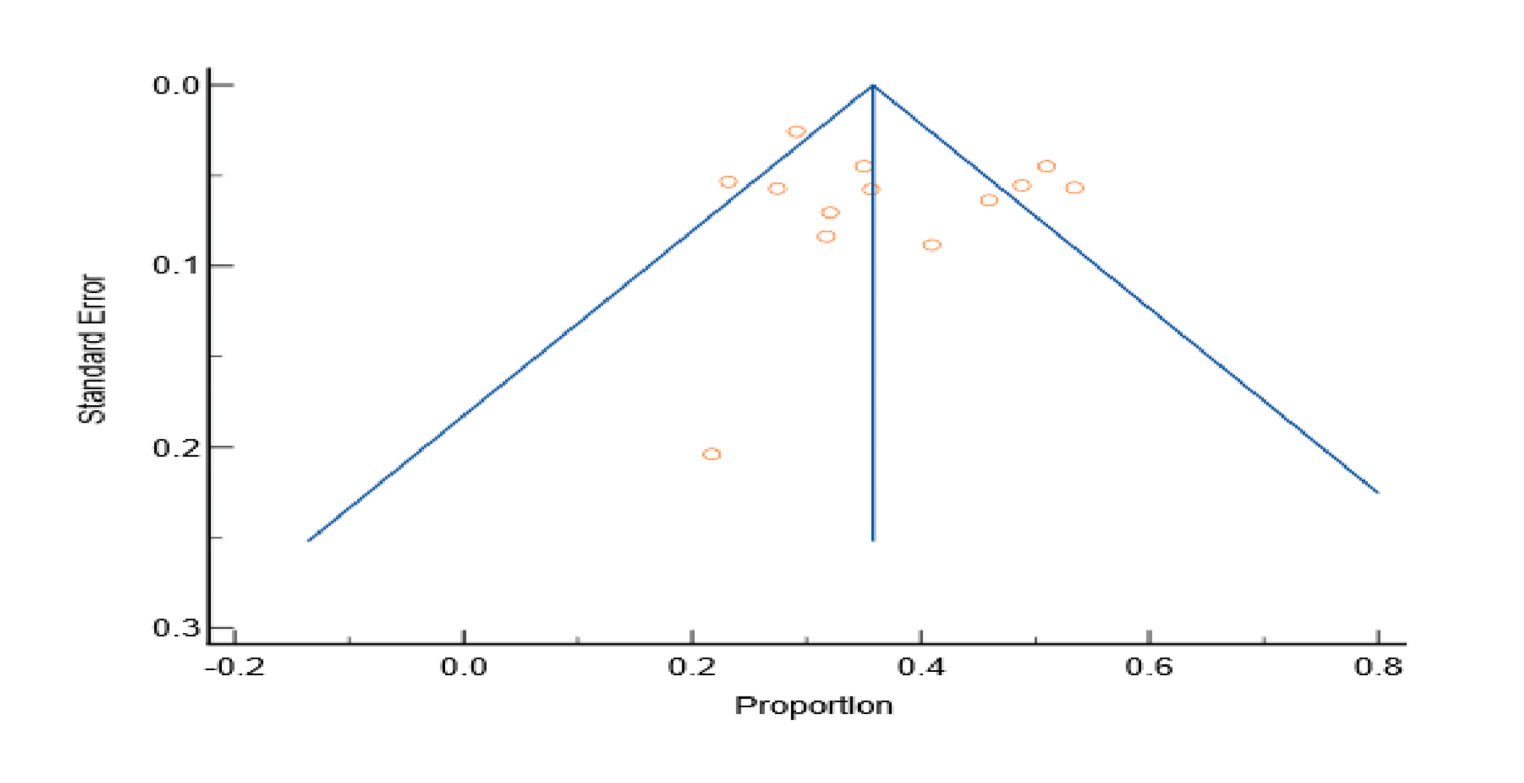
Funnel plot of results of the prevalence of hypertension in patients with type 2 diabetes mellitus patients in Yemen. The image was prepared by author Dr. Zahid Khan using RevMan software.

Of the 19 studies included in our study, only 13 studies [3-6, 9,10,15-17,21,27,35,44] reported the prevalence of hypertension among patients with DM in Yemen. The probability of bias in the results of these studies by the funnel diagram and Egger’s test at the significant level of 0.1 indicated that there is no bias. The total sample size of studies that reported the prevalence of hypertension was 4869 and ranged from 23 to 1540. The lowest reported prevalence of hypertension in these studies was 21.7% by Al-Habori et al. [31], and the highest prevalence was 53.4% in another study [33]. According to the meta-analysis, the prevalence of hypertension in patients with type 2 DM in Yemen was estimated to be 36.9% (Figures 4-5).

Prevalence of smoking

**Figure 6:**
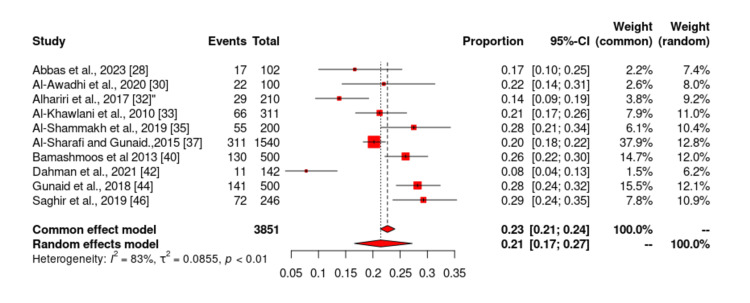
Prevalence of smoking among type 2 diabetes mellitus patients in Yemen. The red boxes represent the effect estimates (prevalence) provided as a percentage. The diamond is the pooled effect estimate at a 95% confidence interval. The image was prepared by author Dr. Zahid Khan using RevMan software.

**Figure 7:**
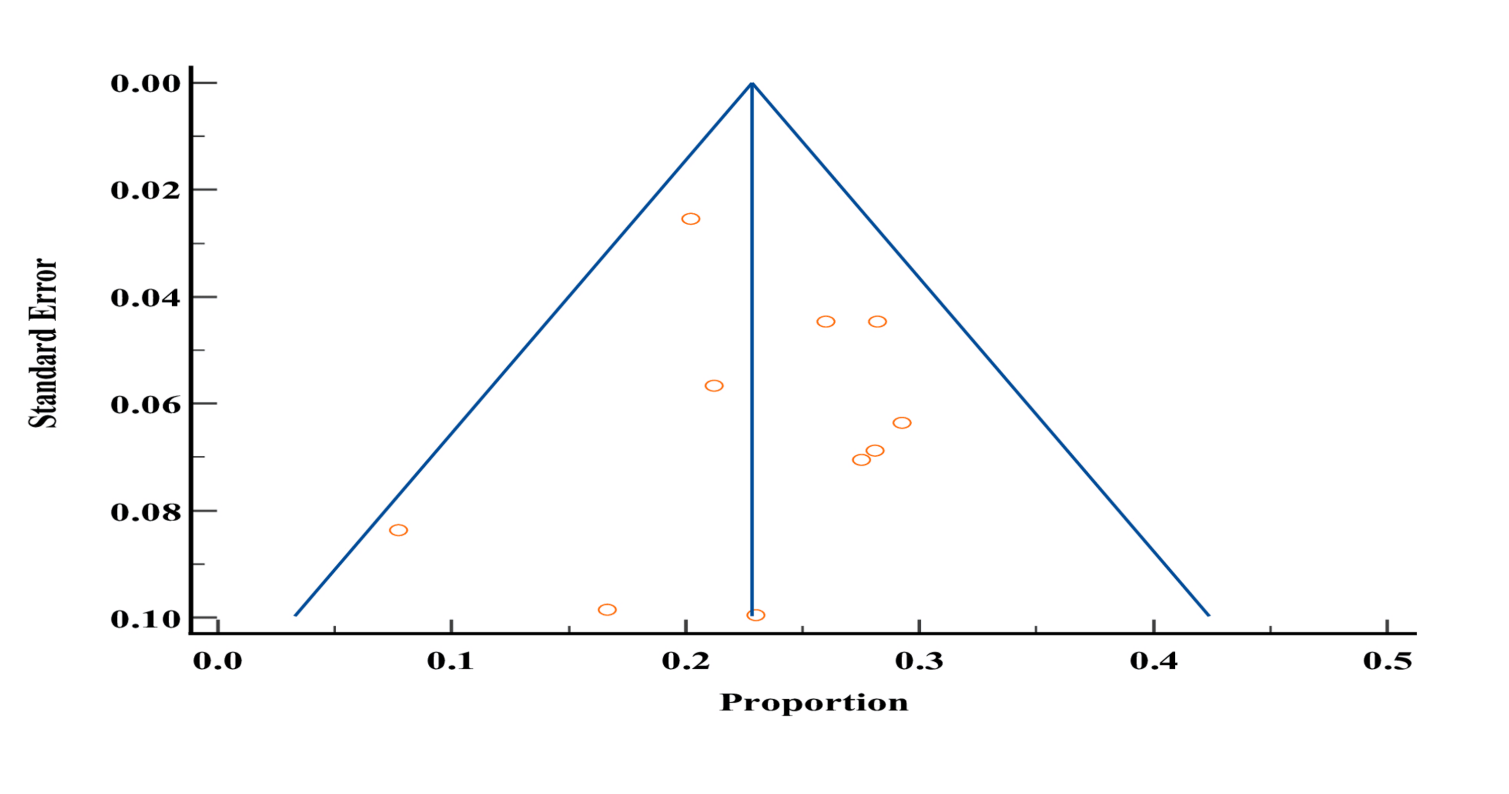
Funnel plot of results of the prevalence of smoking in patients with type 2 diabetes patients in Yemen. The image was prepared by author Dr. Zahid Khan using RevMan software.

Of the 19 studies included in our study, only 10 studies [1,2,5,6,9,11,16,21,27,44] reported the prevalence of smoking among patients with DM in Yemen. The total sample size of studies that reported the prevalence of smoking was 3851 and ranged from 100 to 1540. The lowest reported prevalence of smoking in these studies was 20.2% by Al-Sharafi and Gunaid [37], and the highest prevalence was 29.3% by Saghir et al. [46]. According to the meta-analysis, the prevalence of smoking in patients with type 2 DM in Yemen was estimated to be 22.8% (Figures 6-7). The probability of bias in these studies was pretty low as shown by the funnel diagram (Figure 7).

Prevalence of obesity

**Figure 8:**
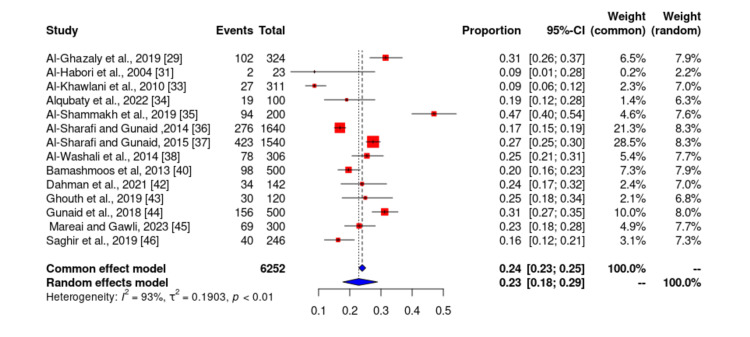
Prevalence of obesity among diabetes melitus patients in Yemen. The red boxes represent the effect estimates (prevalence) provided as a percentage. The diamond is the pooled effect estimate at a 95% confidence interval. The image was prepared by author Dr. Zahid Khan using RevMan software.

**Figure 9:**
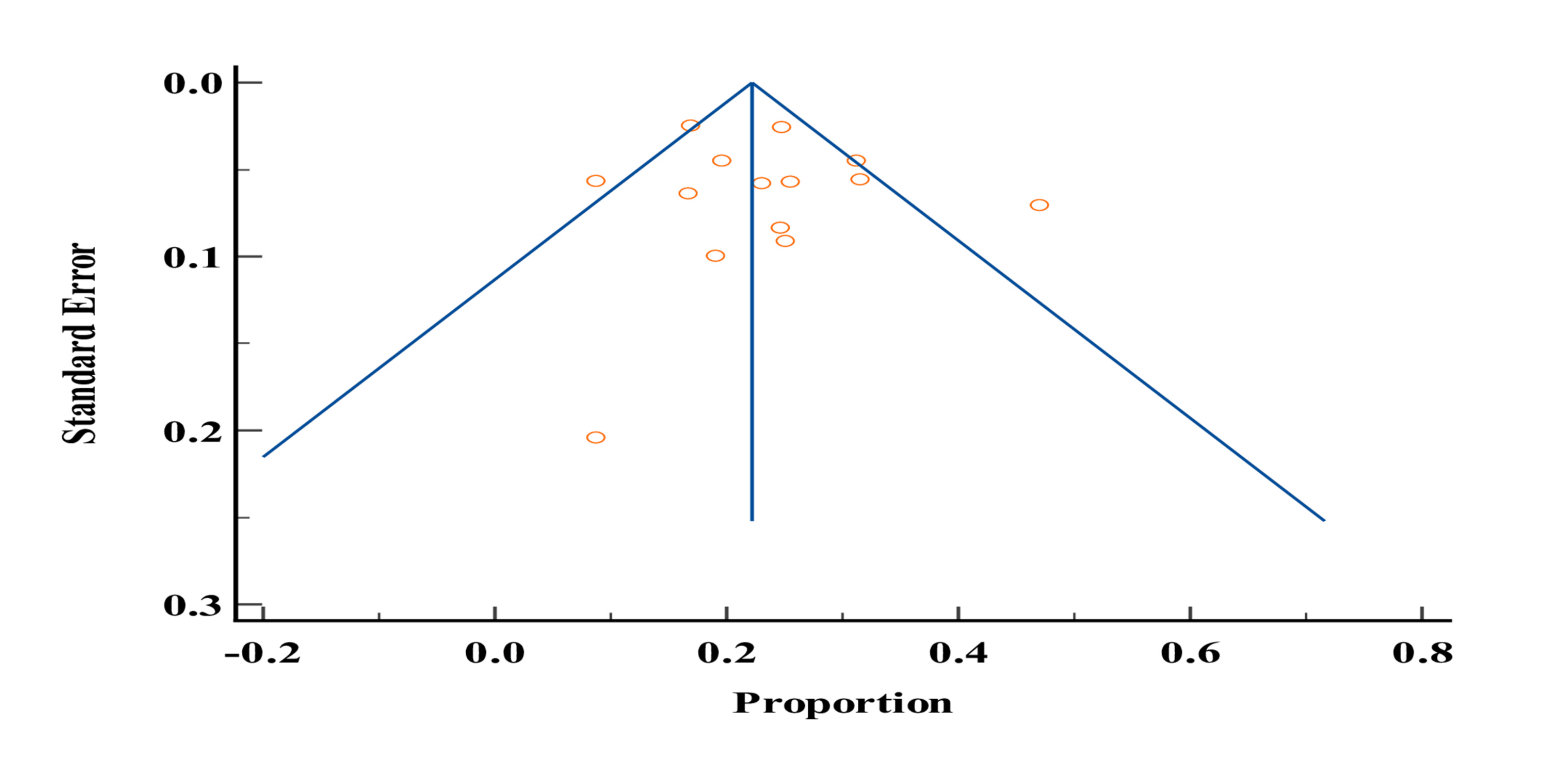
Funnel plot of results of the prevalence of obesity in patients with type 2 diabetes mellitus patients in Yemen. The image was prepared by author Dr. Zahid Khan using RevMan software.

Of the 19 studies included in our study, 14 studies [3-7,9,11,12,16,21,24,27,37,46] reported the prevalence of obesity among patients with DM in Yemen. The total sample size of studies that reported the prevalence of obesity was 6252 and ranged from 23 to 1640. The lowest reported prevalence of obesity in these studies was 8.68% by Al-Khawlani et al. [33] and the highest prevalence was 31.2% by Gunaid et al. [44]. According to the meta-analysis, the prevalence of obesity in patients with type 2 DM in Yemen was estimated to be 23.06% and the probability of publication bias was pretty low (Figures 8-9).

Prevalence of hyperlipidemia

**Figure 10:**
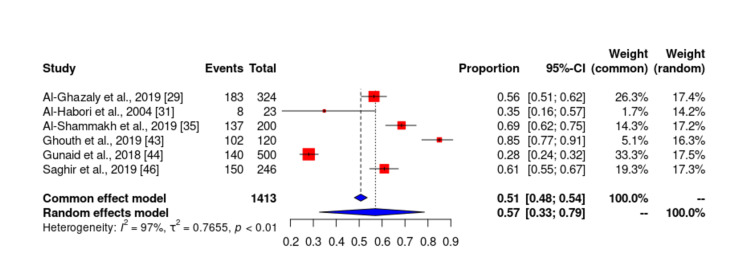
Prevalence of hyperlipidemia among diabetes mellitus patients in Yemen. The red boxes represent the effect estimates (prevalence) provided as a percentage. The diamond is the pooled effect estimate at a 95% confidence interval. The image was prepared by author Dr. Zahid Khan using RevMan software.

**Figure 11:**
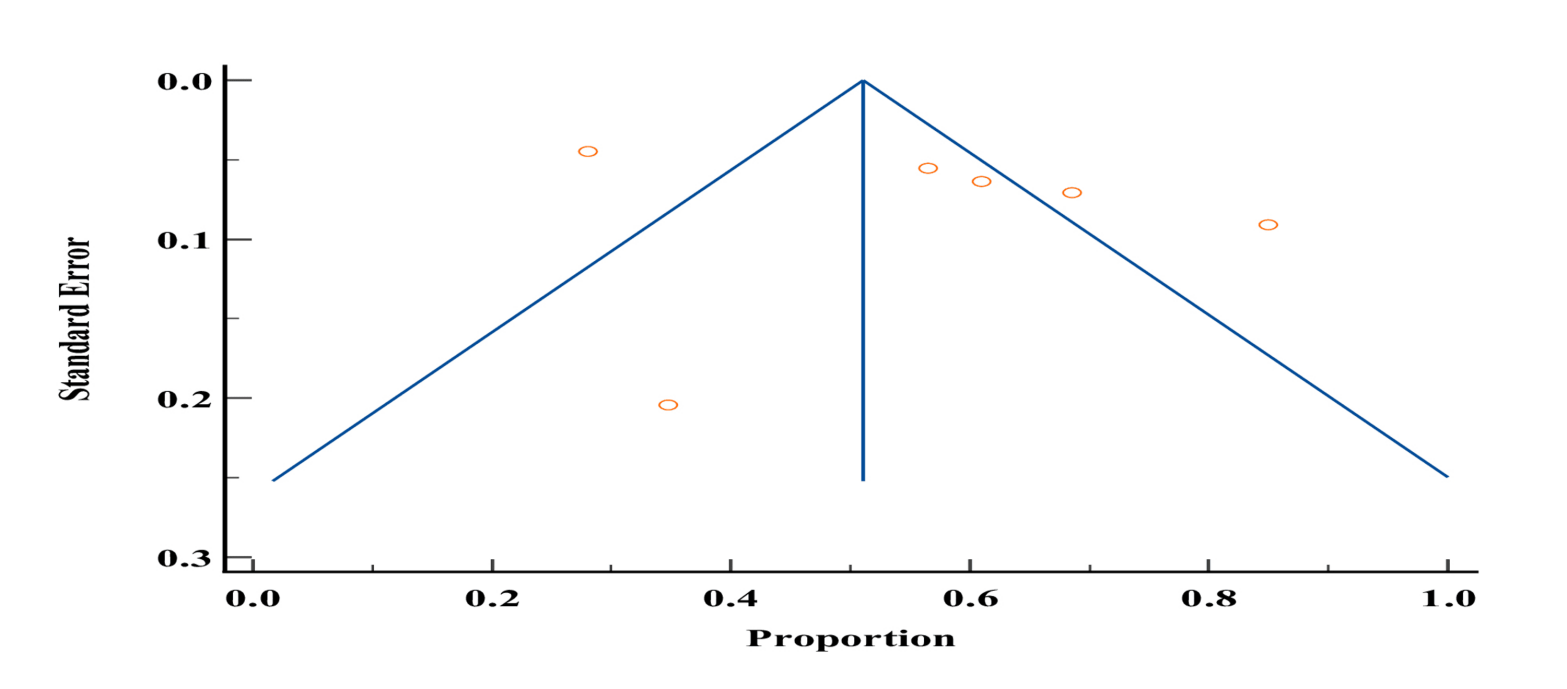
Funnel plot of results of the prevalence of hyperlipidemia in patients with type 2 diabetes patients in Yemen. The image was prepared by author Dr. Zahid Khan using RevMan software.

Of the 19 studies included in our study, only six studies [3,4,6,24,27,46] reported the prevalence of hyperlipidemia among patients with DM in Yemen. The total sample size of studies that reported the prevalence of hyperlipidemia was 1413 and ranged from 23 to 500. The lowest reported prevalence of hyperlipidemia in these studies was 28% by Gunaid et al. [27] and the highest prevalence was 85% by Ghouth et al. [43]. According to the meta-analysis, the prevalence of hyperlipidemia in patients with type 2 DM in Yemen was estimated to be 56.57% (Figures 10-11). The probability of bias in the results of these studies was low as shown by the funnel diagram.

Prevalence of family history

**Figure 12:**
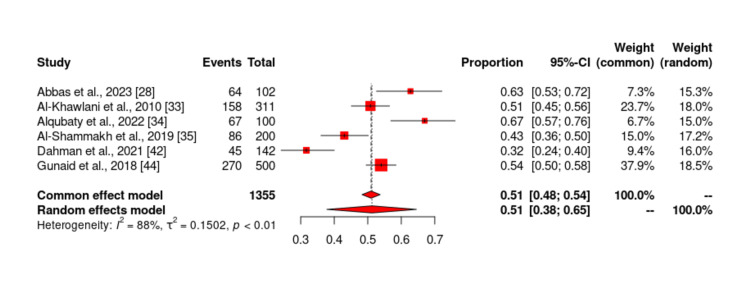
Forest plot showing the prevalence of family history among diabetes mellitus patients in Yemen. The red boxes represent the effect estimates (prevalence) provided as a percentage. The diamond is the pooled effect estimate at a 95% confidence interval. The image was prepared by author Dr. Zahid Khan using RevMan software.

**Figure 13:**
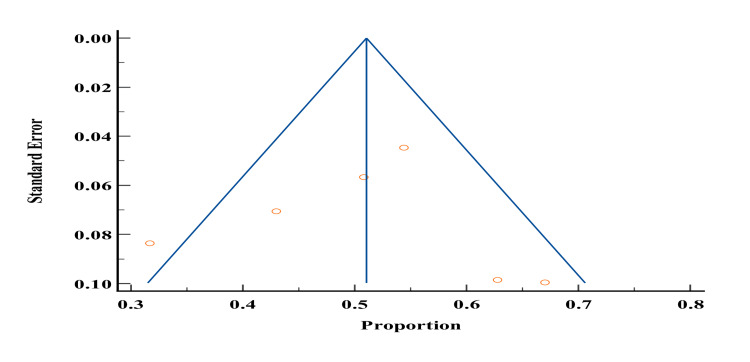
Funnel plot of results of the prevalence of family history in patients with type 2 diabetes mellitus patients in Yemen. The image was prepared by author Dr. Zahid Khan using RevMan software.

Of the 19 studies included in our study, only six studies [1,5,6,12,21,27] reported the prevalence of family history among patients with DM in Yemen. The probability of bias in the results of these studies by the funnel diagram showed no risk of bias. The total sample size of studies that reported the prevalence of family history was 1355 and ranged from 100 to 500. The lowest reported prevalence of family history in these studies was 31.69% by Dahman et al. [42] and the highest prevalence was 67% by Alqubaty et al. [34]. According to the meta-analysis, the prevalence of family history in patients with type 2 DM in Yemen was estimated to be 51.3% (Figures 12-13).

Discussion

The American Heart Association (AHA) has officially listed diabetes, dyslipidemia, hypertension, and smoking as the major risk factors for CHD [47]. A 7-year finish study published by the New England Journal of Medicine suggested that diabetic patients have a similar risk of CHD just like patients with a history of myocardial infarction [47]. Obesity, smoking, hyperlipidemia, hypertension, and other CVR factors are commonly clustered in patients with diabetes. Therefore, management strategies for CHD have been proposed based on metabolic risk factors [48]. This review aimed to systematically determine the prevalence of CV risk factors (hypertension, smoking, obesity, hyperlipidemia, and family history) among individuals with DM in Yemen.

According to this meta-analysis, the prevalence of hypertension in patients with type 2 DM in Yemen was estimated to be 36.9%. The lowest reported prevalence of hypertension in these studies was 21.7%) by Al-Habori et al. [31], and the highest prevalence was reported by Al-Khawlani et al. as 53.4% [33].

This matches with the global trends in The World Health Organization (WHO) Global Report in 2023, which shows that the global prevalence of hypertension remains high, with an estimated 1.28 billion adults aged 30-79 years affected worldwide. A total of 32% of adults aged 30-79 years globally have hypertension, with 34% in men and 32% in women [49].

Traditional Yemeni diets may contribute to hypertension, especially with high salt consumption. The transition toward more processed foods due to urbanization and globalization has introduced unhealthy dietary habits, increasing the risk of hypertension [50]. Lower educational levels and income in Yemen can limit access to healthcare and health information, contributing to higher rates of hypertension. Individuals with lower educational attainment often have poorer health literacy, affecting their ability to manage health conditions effectively [51].

There was a correlation between fluctuations in hypertension prevalence among regions and shifts in rates of awareness, treatment, and control. Indeed, the rate of population coverage for healthcare in Yemen is inadequate, and considerable barriers to accessing health facilities include considerable distances. When traveling to the capital from rural regions of the nation [52]. The results of the present meta-analysis show that the prevalence of smoking in patients with type 2 DM in Yemen was estimated to be 22.8% with the lowest reported prevalence of 20.2% by Al-Sharafi and Gunaid [36] and the highest prevalence was 29.3% by Saghir et al. [46]. Retrospective research conducted by Janousek et al. [53] at a single hospital in Sana’a including 386 patients who had an acute myocardial infarction (AMI) and ranged in age from 27 to 82 years, with the majority being male (90%), revealed a very high incidence of smoking among these patients (81%).

An additional cross-sectional by Alwabr [54] investigation was undertaken including 450 rural women, aged between 18 and 60, who were in attendance at the health centers designated for that purpose in the governorates of Sana’a and Al-Mahweet at the time the research was done. Smoking was shown to be substantially connected with obesity but unrelated to hypertension and DM, according to this research. Furthermore, ambient tobacco smoke exposure (secondhand smoking) causes a significant cardiovascular health risk. There is a 25-30 percent increase in the chance of getting CVDs due to secondhand smoking [55].

This meta-analysis demonstrated that the meta-analysis in our hands showed that the lowest reported prevalence of obesity in these studies was 8.68% by Al-Khawlani et al. [33] and the highest prevalence was 31.2% by Gunaid et al. [44]. According to the meta-analysis, the prevalence of obesity in patients with type 2 DM in Yemen was estimated to be 23.06%. The global incidence of obesity has escalated substantially in recent decades, now approaching epidemic proportions. The WHO has calculated that the prevalence of obesity among adults has escalated by over sevenfold over the last four decades [56]. In a study by Menke et al. [57], they reported that among the three covariates examined (age, race/ethnicity, and BMI), an analysis spanning more than three decades determined that the increase in BMI over time was the most significant concerning the rise in diabetes prevalence. Specifically, it accounted for around 50 percent of the increase in diabetes prevalence among males and 100 percent among females. Furthermore, nurses with a baseline BMI greater than or equal to 35 kg/m^2^ had an almost 100-fold higher chance of developing incident diabetes throughout 14 years, compared to those with a BMI less than or equal to 22 kg/m^2^ [58].

The obesogenic environment, which stimulates overnutrition, leads to dysregulation of metabolic balance and subsequent fat accumulation in organs not specialized for lipid storage, such as the endothelium, liver, and skeletal muscle, inducing metabolic disturbances and disorders. Several mechanisms are strictly linked to the onset of CVD and atherosclerosis, among which endothelial dysfunction plays a major role [59]. The interplay between obesity, severe obesity, insulin resistance, chronic inflammation, and the development of cardiac diseases has been highlighted [58]. Obesity is a recognized risk factor for heart failure (HF), CHD, and premature death. CVD risk factors related to obesity, such as high cholesterol and blood pressure, have a high prevalence in overweight and obese people, even though the increased use of medications has reduced risk factors for CVD and improved disease management [60].

Based on a systematic review and meta-analysis estimates in 2020, the prevalence of obesity among those aged 18 and above in Yemen was 8.8% and the prevalence of overweight was 23.5% [61]. A study conducted among persons aged 15-69 years, including both urban and rural populations, had comparable results: women had a higher prevalence of obesity, abdominal obesity, and elevated cholesterol compared to males. The prevalence of obesity was found to be greater among urban individuals in comparison to their rural counterparts [61]. Overweight prevalence was higher among males than females (18% vs 14%). While 12.4% of females and only 2.5% of males were obese [36].

The data from this meta-analysis showed that the lowest reported prevalence of hyperlipidemia in these studies was 28% by Gunaid et al. [44] and the highest prevalence was 85% by Ghouth et al. [43]. According to the meta-analysis, the prevalence of hyperlipidemia in patients with type 2 DM in Yemen was estimated to be 56.57%.

A range of 70% to 97% of adults with type 2 diabetes have one or more lipid abnormalities, this is called diabetic dyslipidemia or atherogenic dyslipidemia. Diabetic dyslipidemia is characterized by elevated triglycerides, low high-density lipoprotein levels, and the presence of smaller and denser low-density lipoprotein particles [62]. Another study by Dayakar et al. [63] reported that 58.6% of T2DM patients had hypercholesterolemia and 36.9% had hypertriglyceridemia, with increased low-density lipoprotein (LDL) levels observed in 65.2% of patients. Early identification of dyslipidemia, a well-acknowledged and modifiable risk factor, is crucial to implementing proactive cardiovascular preventive measures. As a result of lipid alterations, patients with type 2 DM have an increased risk of developing vascular illnesses. A comprehensive examination of lipid problems and insulin administration in diabetics [64].

Our findings indicated that the lowest reported prevalence of family history in these studies was 31.69% by Dahman et al. [42] and the highest prevalence was 67% by Alqubaty et al. [34]. According to the meta-analysis, the prevalence of family history in patients with type 2 DM in Yemen was estimated to be 51.3%. Extensive genome-wide association studies have identified over 500 different genetic signals that are strongly correlated with type 2 diabetes. Among them, one of the biggest multi-ancestral meta-analyses, which included 1.4 million human participants, identified over 300 of these signals [65]. In a case-control study carried out from November 2018 to May 2019 on diabetic patients with or without foot ulcers, the prevalence of family history was 55% [66].

The results of a survey that was carried out in the Hamdan area of Yemen, specifically among persons who were at least 35 years old, revealed that the prevalence of diabetes and hypertension in first-degree relatives was found to be frequent among the study sample. Compared to those who have a negative family history, the likelihood of this occurring is about 2.4 times higher. It has been shown that children who are born to parents who have diabetes and who are either consanguineous or conjugal are more likely to acquire type 2 diabetes at an earlier age [67]. A positive family history is a significant risk factor for getting CVD that cannot be changed. It is general knowledge that having a family history of a common chronic illness may raise the likelihood of obtaining these diseases by a factor of two to five, and this likelihood rises in proportion to the number of relatives who are afflicted by the disease [68]. The research discovered a connection between high glucose levels and CVD. Using the data from 59 additional genetic variations, DM was shown to be related to CVD as having an odds ratio of 1.63. There were nine of these variations that had an odds ratio of 1.53 for every one percent rise in HbA1C to the increased risk of CVD, which suggests that there is a causal association between type 2 diabetes and CVD on a genetic level [69]. Whether the relationship between diabetes and CVD is attributable to diabetes status itself or the risk factors that diabetic people are more likely to be exposed to is still a matter of debate [70].

## Conclusions

Our study revealed that hypertension, smoking, obesity, hyperlipidemia, and family history were prevalent among individuals with type 2 DM in Yemen. The estimated prevalence rates were 36.9% for hypertension, 22.8% for smoking, 23.06% for obesity, and 56.57% for hyperlipidemia. The study also highlighted the regional distribution of these risk factors, with a significant number of studies conducted in the capital city of Sana’a. Overall, these findings contribute to a better understanding of the evolving cardiovascular landscape within the Yemeni population.

## References

[1] Abbas AB, Hazeb A, Al-Badani R (2023). A case-control study to evaluate hematological indices in blood of diabetic and non-diabetic individuals in Ibb City, Yemen. Sci Rep.

[2] United Nations (2022 (2024). United Nations. Department of economic and social affairs: Population division. https://population.un.org/wpp/Download/Standard/MostUsed/.

[3] United Nations (2019 (2024). Human development report 2019. https://hdr.undp.org/content/human-development-report-2019.

[4] GBD 2019 Diseases and Injuries Collaborators (2020). Global burden of 369 diseases and injuries in 204 countries and territories, 1990-2019: A systematic analysis for the Global Burden of Disease Study 2019. Lancet.

[5] Patel DK, Kumar R, Laloo D, Hemalatha S (2012). Diabetes mellitus: An overview on its pharmacological aspects and reported medicinal plants having antidiabetic activity. Asian Pac J Trop Biomed.

[6] Craig ME, Hattersley A, Donaghue KC (2009). Definition, epidemiology and classification of diabetes in children and adolescents. Pediatr Diabetes.

[7] Tan SY, Mei Wong JL, Sim YJ (2019). Type 1 and 2 diabetes mellitus: A review on current treatment approach and gene therapy as potential intervention. Diabetes Metab Syndr.

[8] Abdulah DM, Hassan AB, Saadi FS, Mohammed AH (2018). Impacts of self-management education on glycaemic control in patients with type 2 diabetes mellitus. Diabetes Metab Syndr.

[9] Ogurtsova K, da Rocha Fernandes JD, Huang Y (2017). IDF Diabetes Atlas: Global estimates for the prevalence of diabetes for 2015 and 2040. Diabetes Res Clin Pract.

[10] Haile TG, Mariye T, Tadesse DB, Gebremeskel GG, Asefa GG, Getachew T (2023). Prevalence of hypertension among type 2 diabetes mellitus patients in Ethiopia: A systematic review and meta-analysis. Int Health.

[11] www.who.int. (2013 (2024). A global brief on hypertension: Silent killer, global public health crisis: World Health Day 2013. World Health Day.

[12] Wake AD (2023). Incidence and predictors of hypertension among diabetic patients attending a diabetic follow-up clinic in Ethiopia: A retrospective cohort study. J Int Med Res.

[13] Benowitz NL, Liakoni E (2022). Tobacco use disorder and cardiovascular health. Addiction.

[14] Fu M, Mei A, Min X (2024). Advancements in cardiovascular disease research affected by smoking. Rev Cardiovasc Med.

[15] Kondo T, Nakano Y, Adachi S, Murohara T (2019). Effects of tobacco smoking on cardiovascular disease. Circ J.

[16] Benowitz NL, Burbank AD (2016). Cardiovascular toxicity of nicotine: Implications for electronic cigarette use. Trends Cardiovasc Med.

[17] Sarma S, Sockalingam S, Dash S (2021). Obesity as a multisystem disease: Trends in obesity rates and obesity-related complications. Diabetes Obes Metab.

[18] Powell-Wiley TM, Poirier P, Burke LE (2021). Obesity and cardiovascular disease: A scientific statement from the American Heart Association. Circulation.

[19] La Sala L, Prattichizzo F, Ceriello A (2019). The link between diabetes and atherosclerosis. Eur J Prev Cardiol.

[20] Poznyak A, Grechko AV, Poggio P, Myasoedova VA, Alfieri V, Orekhov AN (2020). The diabetes mellitus-atherosclerosis connection: The role of lipid and glucose metabolism and chronic inflammation. Int J Mol Sci.

[21] Berberich AJ, Hegele RA (2022). A modern approach to dyslipidemia. Endocr Rev.

[22] Albache N, Al Ali R, Rastam S, Fouad FM, Mzayek F, Maziak W (2010). Epidemiology of type 2 diabetes mellitus in Aleppo, Syria. J Diabetes.

[23] Scott RA, Langenberg C, Sharp SJ (2013). The link between family history and risk of type 2 diabetes is not explained by anthropometric, lifestyle or genetic risk factors: The EPIC-InterAct study. Diabetologia.

[24] Meigs JB, Cupples LA, Wilson PW (2000). Parental transmission of type 2 diabetes: The Framingham Offspring Study. Diabetes.

[25] Groop L, Forsblom C, Lehtovirta M (1996). Metabolic consequences of a family history of NIDDM (the Botnia study): Evidence for sex-specific parental effects. Diabetes.

[26] Lo CK, Mertz D, Loeb M (2014). Newcastle-Ottawa Scale: Comparing reviewers' to authors' assessments. BMC Med Res Methodol.

[27] Herzog R, Álvarez-Pasquin MJ, Díaz C, Del Barrio JL, Estrada JM, Gil Á (2013). Are healthcare workers' intentions to vaccinate related to their knowledge, beliefs and attitudes? A systematic review. BMC Public Health.

[28] Abbas AB, Hazeb A, Al-Badani R (2023). A case-control study to evaluate hematological indices in blood of diabetic and non-diabetic individuals in Ibb City, Yemen. Sci Rep.

[29] Al-Ghazaly J, Atef Z, Al-Dubai W (2019). Pattern and causes of anemia in Yemeni patients with type 2 diabetes mellitus. Eur J Biomed Pharm Sci.

[30] Al-Awadhi EF, Bahaj S, Al-Oferi B, Esmail A, Al-Arnoot S (2020). Helicobacter pylori infection among patients with type II diabetes mellitus. J Diabetes Metab Disord Control.

[31] Al-Habori M, Al-Mamari M, Al-Meeri A (2004). Type II diabetes mellitus and impaired glucose tolerance in Yemen: prevalence, associated metabolic changes and risk factors. Diabetes Res Clin Pract.

[32] Alhariri A, Daud F, Almaiman A, Saghir S (2017). Factors associated with adherence to diet and exercise among type 2 diabetes patients in Hodeidah city, Yemen. Life.

[33] Al-Khawlani A, Atef ZA, Al-Ansi A (2010). Macrovascular complications and their associated risk factors in type 2 diabetic patients in Sana'a city, Yemen. East Mediterr Health J.

[34] Alqubaty Alqubaty, A A, Alhaj Alhaj, A A, Al-qadasi Al-qadasi, F F (2022). Serum electrolyte levels among patients with type 2 diabetes mellitus in Sana’a City, Yemen. Zagazig Univ Med J.

[35] Al-Shammakh AA, Ali AD, Al Jermozy H (2019). Prevalence of Proteinuria among type 2 diabetic patients in Dhamar Governorate, Yemen. Int J Diabetes Clin Res.

[36] Al-Sharafi BA, Gunaid AA (2014). Prevalence of obesity in patients with type 2 diabetes mellitus in yemen. Int J Endocrinol Metab.

[37] Al-Sharafi BA, Gunaid AA (2015). Effect of habitual khat chewing on glycemic control, body mass index, and age at diagnosis of diabetes in patients with type 2 diabetes mellitus in Yemen. Clin Med Insights Endocrinol Diabetes.

[38] Al Washali AY, Ariffin AA, Hejar AR, Amani YW (2014). Prevalence and associated risk factors of diabetic peripheral neuropathy among diabetic patients in national center of diabetes in Yemen. Int J Public Health Clin Sci.

[39] Bamahel A, Bafakeer S, Bajaber E (2022). Prevalence of diabetic nephropathy among type 2 diabetes mellitus patients in Mukalla City, Yemen. EKST.

[40] Bamashmoos M, Bamashmoos M, Ganem Y (2013). Diabetic nephropathy and its risk factors in type 2-diabetic patients in Sana’a City, Yemen. World J Med Sci.

[41] Bamashmus MA, Gunaid AA, Khandekar RB (2009). Diabetic retinopathy, visual impairment and ocular status among patients with diabetes mellitus in Yemen: A hospital-based study. Indian J Ophthalmol.

[42] Dahman LS, Humam MA, Musiaan NS, Daakik AM, Balfas MA (2021). Elevated liver enzymes and its association with type two diabetes mellitus: The occurrence in Yemeni population. Int J Endocrinol Metab Disord.

[43] Ghouth AS, Ba-Karman AA, Alaidroos HA (2019). Prevalence and patterns of dyslipidemia among type2 diabetes mellitus patients in Mukalla City ,Yemen, in 2017. Community Med Public Health Care.

[44] Gunaid AA, Al-Kebsi MM, Bamashmus MA, Al-Akily SA, Al-Radaei AN (2018). Clinical phenotyping of newly diagnosed type 2 diabetes in Yemen. BMJ Open Diabetes Res Care.

[45] Mareai SS, Gawli K (2023). Type 2 diabetes with obesity and hypertension: Prevalence and sociodemographic risk factors in Yemen. Diabetes mellitus.

[46] Saghir SA, Alhariri AE, Alkubat SA, Almiamn AA, Aladaileh SH, Alyousefi NA (2019). Factors associated with poor glycemic control among type-2 diabetes mellitus patients in Yemen. Trop J Pharm Res.

[47] Wang C, Ye D, Xie Z (2021). Assessment of cardiovascular risk factors and their interactions in the risk of coronary heart disease in patients with type 2 diabetes with different weight levels, 2013-2018. Diabetes Metab Syndr Obes.

[48] Chung WK, Erion K, Florez JC (2020). Precision medicine in diabetes: A consensus report from the American Diabetes Association (ADA) and the European Association for the study of diabetes (EASD). Diabetes Care.

[49] Kario K, Okura A, Hoshide S, Mogi M (2024). The WHO Global report 2023 on hypertension warning the emerging hypertension burden in globe and its treatment strategy. Hypertens Res.

[50] Modesti PA, Bamoshmoosh M, Rapi S, Massetti L, Al-Hidabi D, Al Goshae H (2013). Epidemiology of hypertension in Yemen: Effects of urbanization and geographical area. Hypertens Res.

[51] Solomon M, Shiferaw BZ, Tarekegn TT (2023). Prevalence and associated factors of hypertension among adults in gurage zone, Southwest Ethiopia, 2022. SAGE Open Nurs.

[52] Gunaid AA (2002). Prevalence of known diabetes and hypertension in the Republic of Yemen. East Mediterr Health J.

[53] Janousek S, Al-Kubati M, Al-Shwafi KA (2008). Risk factors, clinical features and outcome of acute myocardial infarction in Sana'a, Yemen. Ann Saudi Med.

[54] Alwabr GMA (2018). Prevalence of risk factors for noncommunicable diseases among rural women in Yemen. Fam Med Community Health.

[55] Nasser AM, Salah BA, Regassa LT, Alhakimy AA, Zhang X (2018). Smoking prevalence, attitudes and associated factors among students in health-related Departments of Community College in rural Yemen. Tob Induc Dis.

[56] NCD Risk Factor Collaboration (NCD-RisC) (2017). Worldwide trends in body-mass index, underweight, overweight, and obesity from 1975 to 2016: A pooled analysis of 2416 population-based measurement studies in 128·9 million children, adolescents, and adults. Lancet.

[57] Menke A, Rust KF, Fradkin J, Cheng YJ, Cowie CC (2014). Associations between trends in race/ethnicity, aging, and body mass index with diabetes prevalence in the United States: A series of cross-sectional studies. Ann Intern Med.

[58] Zhao M, Bovet P, Xi B (2020). Weight status change from adolescence to young adulthood and the risk of hypertension and diabetes mellitus. Hypertension.

[59] Volpe M, Gallo G (2023). Obesity and cardiovascular disease: An executive document on pathophysiological and clinical links promoted by the Italian Society of Cardiovascular Prevention (SIPREC). Front Cardiovasc Med.

[60] Unamuno X, Gómez-Ambrosi J, Rodríguez A, Becerril S, Frühbeck G, Catalán V (2018). Adipokine dysregulation and adipose tissue inflammation in human obesity. Eur J Clin Invest.

[61] Okati-Aliabad H, Ansari-Moghaddam A, Kargar S, Jabbari N (2022). Prevalence of obesity and overweight among adults in the Middle East Countries from 2000 to 2020: A systematic review and meta-analysis. J Obes.

[62] Bahiru E, Hsiao R, Phillipson D, Watson KE (2021). Mechanisms and treatment of dyslipidemia in diabetes. Curr Cardiol Rep.

[63] Dayakar E, Sree CS, Sanjay E (2019). Study on the prevalence of dyslipidemia in type 2 diabetes mellitus. IJAM.

[64] Wiggins BS, Dixon D, Bellone J, Gasbarro N, Marrs JC, Tran R (2020). Key articles and guidelines in the management of dyslipidemia: 2019 Update. J Pharm Pract.

[65] Vujkovic M, Keaton JM, Lynch JA (2020). Discovery of 318 new risk loci for type 2 diabetes and related vascular outcomes among 1.4 million participants in a multi-ancestry meta-analysis. Nat Genet.

[66] Hameed B, Baras MH (2020). The risk factors of developing diabetic foot ulcers among diabetic patients in Mukalla City-Hadhramout/Yemen. Sudan JMS.

[67] Gunaid AA, Assabri AM (2008). Prevalence of type 2 diabetes and other cardiovascular risk factors in a semirural area in Yemen. East Mediterr Health J.

[68] Yoon PW, Scheuner MT, Peterson-Oehlke KL, Gwinn M, Faucett A, Khoury MJ (2002). Can family history be used as a tool for public health and preventive medicine?. Genet Med.

[69] Ross S, Gerstein HC, Eikelboom J, Anand SS, Yusuf S, Paré G (2015). Mendelian randomization analysis supports the causal role of dysglycaemia and diabetes in the risk of coronary artery disease. Eur Heart J.

[70] Glovaci D, Fan W, Wong ND (2019). Epidemiology of diabetes mellitus and cardiovascular disease. Curr Cardiol Rep.

